# A Facet-Associated Cyst with Aneurysmal Bone Cyst Morphology Causing T10–T11 Compressive Myelopathy Mimicking Cauda Equina Syndrome in an Octogenarian

**DOI:** 10.3390/diagnostics16203320

**Published:** 2026-10-13

**Authors:** Symeon Naoum, Christopher Brown, Daniel Rolton, George Triantafyllou, Nikolaos-Achilleas Arkoudis, Maria Piagkou, Christos Koutserimpas

**Affiliations:** 1 Department of Trauma and Orthopaedics, Royal Berkshire Hospital, Royal Berkshire NHS Foundation Trust, London Road, Reading, Berkshire RG1 5AN, UK; naoumsimeon@gmail.com (S.N.); christopher.brown@royalberkshire.nhs.uk (C.B.); daniel.rolton@royalberkshire.nhs.uk (D.R.); 2 Department of Anatomy, School of Medicine, Faculty of Health Sciences, National and Kapodistrian University of Athens (UoA), 11527 Athens, Greece; georgerose406@gmail.com (G.T.); piagkoumara@gmail.com (M.P.); 3 2nd Department of Radiology, University General Hospital “ATTIKON”, Medical School, National and Kapodistrian University of Athens, 12462 Athens, Greece; nick.arkoudis@gmail.com; 4 School of Health Rehabilitation Sciences, University of Patras, 26504 Patras, Greece

**Keywords:** aneurysmal bone cyst, thoracic spine, compressive myelopathy, cauda equina syndrome mimic, saddle anesthesia, spinal cord compression, octogenarian

## Abstract

Aneurysmal bone cysts (ABCs) are benign, locally aggressive osseous lesions that predominantly affect children and young adults; ABC morphology in a facet-associated cystic lesion in an octogenarian is highly unusual and may overlap radiologically with a degenerative facet or synovial cyst. An 81-year-old woman presented with a 2-month history of progressive bilateral lower-limb weakness, sensory disturbance, and gait deterioration, followed by acute saddle anaesthesia and bowel dysfunction, prompting concern for cauda equina syndrome. Examination showed bilateral MRC 3/5 weakness, mildly increased lower-limb tone, brisk reflexes, and bilateral Hoffmann signs. Lumbar MRI did not demonstrate cauda equina compression. Dedicated thoracic MRI revealed severe multifactorial T10–T11 canal stenosis with marked cord compression, together with a small right posterolateral extradural lesion adjacent to the facet/posterior elements. Posterior T10–T11 decompression, including laminectomy and flavectomy, with excision of the cystic lesion resulted in neurological improvement. Histopathology demonstrated blood-filled cystic spaces separated by fibrovascular septa containing osteoclast-like multinucleated giant cells and reactive bone, without malignant features, consistent with ABC morphology. This case emphasizes careful neurological localization when cauda equina-like symptoms coexist with upper motor neuron signs and highlights an unusual facet-associated cystic lesion with ABC morphology contributing to multifactorial thoracic spinal stenosis in an elderly patient.

**Figure 1:**
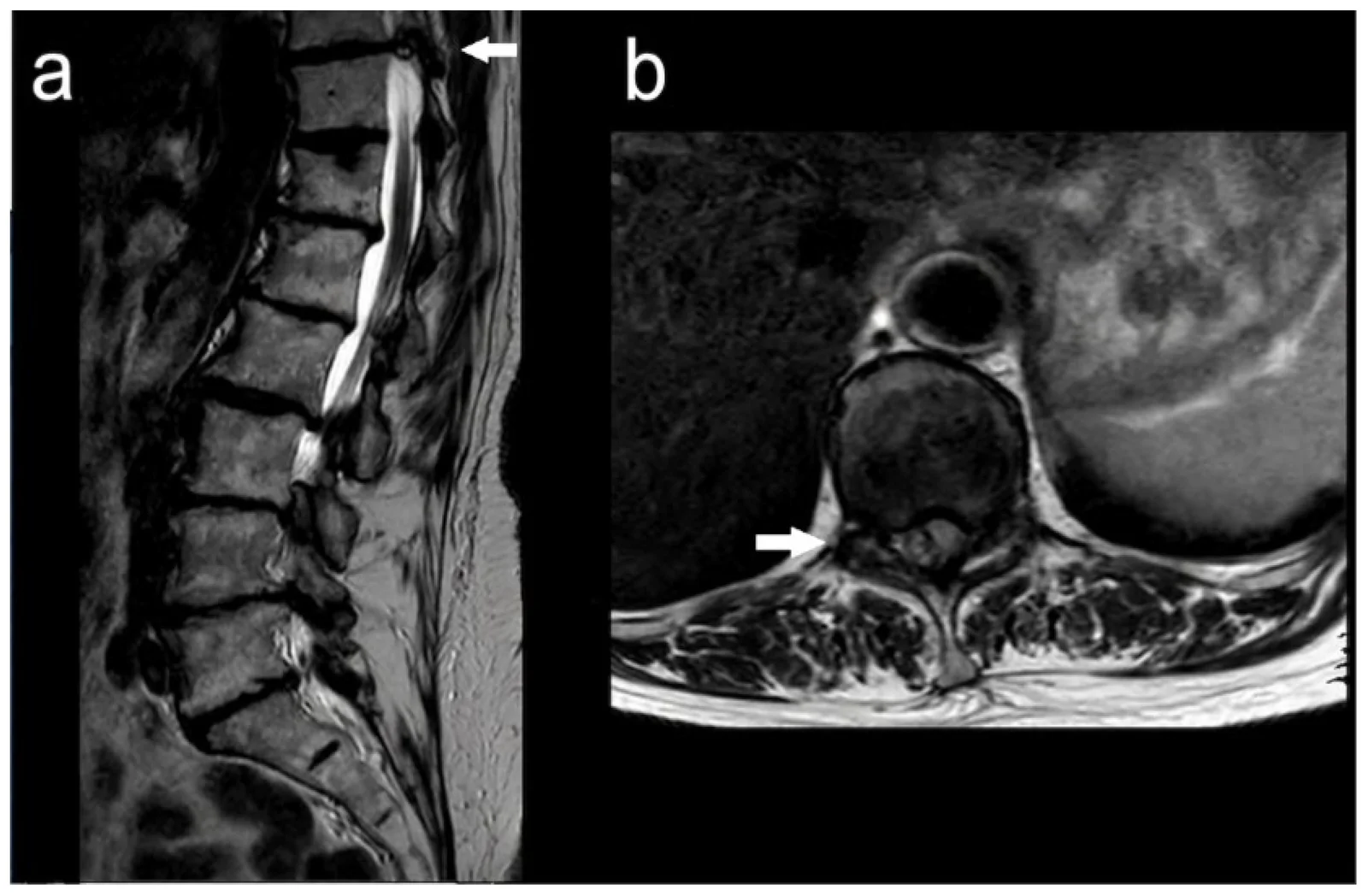
An 81-year-old woman, previously independent and ambulatory without walking aids, presented with a 2-month history of progressive bilateral lower-limb sensory disturbance and weakness accompanied by worsening gait. Two days before presentation, she developed new-onset saddle anaesthesia, worsening constipation, and difficulty with bowel evacuation. She reported no urinary retention, urgency, incontinence, or faecal incontinence. Neurological examination demonstrated symmetrical bilateral lower-limb weakness (MRC 3/5 from L2 to S1), mildly increased tone, non-dermatomal sensory impairment, impaired distal vibration and proprioception, diminished perineal sensation and brisk deep tendon reflexes. Upper-extremity neurological examination demonstrated normal bilateral motor function and no clinically significant upper-limb sensory deficit. Bilateral Hoffmann signs were present. Anal tone was preserved. Post-void bladder scanning showed a residual volume of 203 mL, an elevated value around the commonly used 200-mL risk threshold in suspected cauda equina syndrome (CES), although post-void residual volume should be interpreted only as an adjunct to the overall clinical assessment [1]. The combination of saddle anaesthesia, bowel symptoms, bilateral lower-limb neurological dysfunction, and an elevated post-void residual appropriately raised immediate concern for CES. Current consensus supports urgent magnetic resonance imaging (MRI) when CES is suspected because clinical findings alone cannot reliably confirm or exclude cauda equina compression [2]. However, lower-limb hypertonia and hyperreflexia were discordant with an isolated lumbosacral nerve-root disorder, while bilateral Hoffmann signs further raised concern for a more proximal cord process. Previous brain MRI had demonstrated severe chronic small-vessel ischaemic change without an intracranial mass, acute infarction, or other focal intracranial lesion. Although chronic small-vessel disease may potentially contribute to corticospinal tract dysfunction, no discrete intracranial lesion was identified to account for the Hoffmann signs. Initial lumbar spine MRI was obtained because of the progressive bilateral neurological deficits and new-onset saddle anaesthesia. (**a**) Sagittal T2-weighted imaging from T12 to S1 demonstrates mild levoconvex scoliosis and multilevel degenerative disc and facet-joint disease, a T10–T11 level 5-mm right posterolateral extradural cystic lesion abutting the facet/posterior elements (white arrow) with variable lateral recess and right-sided foraminal narrowing but no significant central canal stenosis or cauda equina compression to account for the bilateral lower-limb weakness, upper motor neuron signs, and saddle sensory disturbance. (**b**) Limited axial T2-weighted imaging through T10–T11, obtained as part of the available lower-spine examination, provides only partial assessment of the lower thoracic canal but demonstrates a small right posterolateral extradural cystic-appearing lesion abutting the right facet/posterior elements (white arrow). Despite its small size, the lesion occupies a functionally important portion of an already narrowed canal and contributes to focal displacement and compression of the distal thoracic spinal cord. The combination of lower-limb upper motor neuron signs and the absence of explanatory lumbar neural compression prompted further imaging of the cervical and thoracic spine. Thoracic spinal cord compression is an uncommon but recognized mimic of cauda equina syndrome [3].

**Figure 2:**
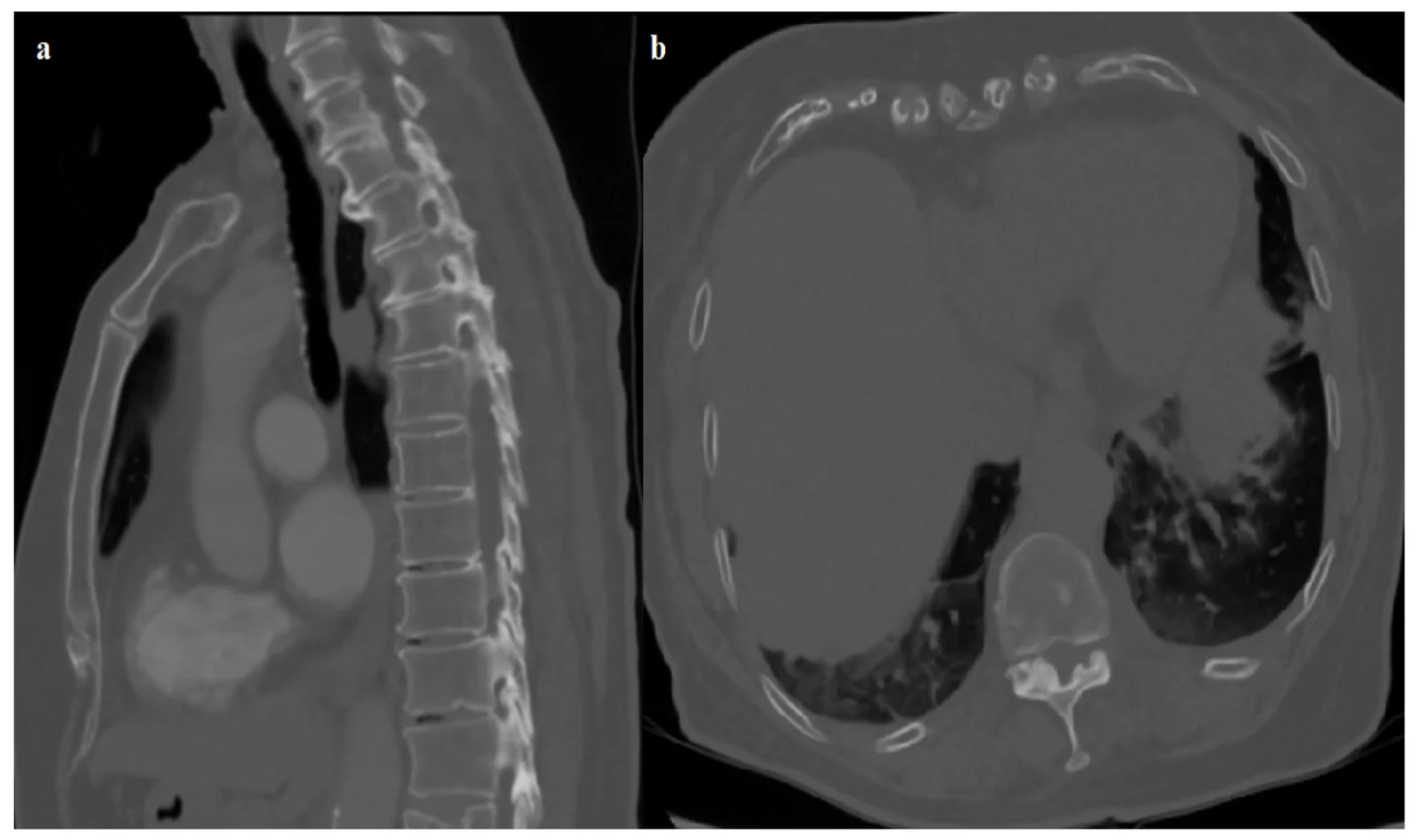
Computed tomography (CT) of the thoracic spine was reviewed to further characterize the lesion identified on the initial MRI, particularly its relationship to the posterior elements and facet joint, and to assess for associated osseous abnormalities. (**a**) Sagittal CT reconstruction demonstrates the T10–T11 level in the context of multilevel degenerative spinal disease, including chronic spondylotic changes and multilevel disc–osteophyte complexes. (**b**) Axial CT imaging at T10–T11 demonstrates focal asymmetry and degenerative remodelling centred on the right facet-joint/posterior-element complex, corresponding to the location of the cystic lesion identified on the initial MRI. There is focal cortical irregularity and remodelling of the adjacent articular processes, without a definite discrete expansile osteolytic lesion, marked cortical thinning, or substantial osseous expansion of the posterior elements. The lesion is closely related to the right T10–T11 facet joint and extends into the right posterolateral aspect of the spinal canal, contributing to local canal narrowing in combination with facet hypertrophy and ligamentum flavum thickening. The remaining posterior elements are preserved without evidence of destructive osseous change. Overall, the CT findings demonstrate a facet-associated cystic lesion with adjacent focal cortical remodelling in the setting of advanced degenerative facet disease, but without definitive imaging evidence of a primary expansile intraosseous lesion. At this stage, a degenerative facet/synovial cyst remained an important differential diagnosis, particularly given the patient’s advanced age, lesion location, and substantial facet degeneration. Further characterization of the spinal canal, spinal cord, and extent of neural compression was therefore undertaken with dedicated thoracic MRI.

**Figure 3:**
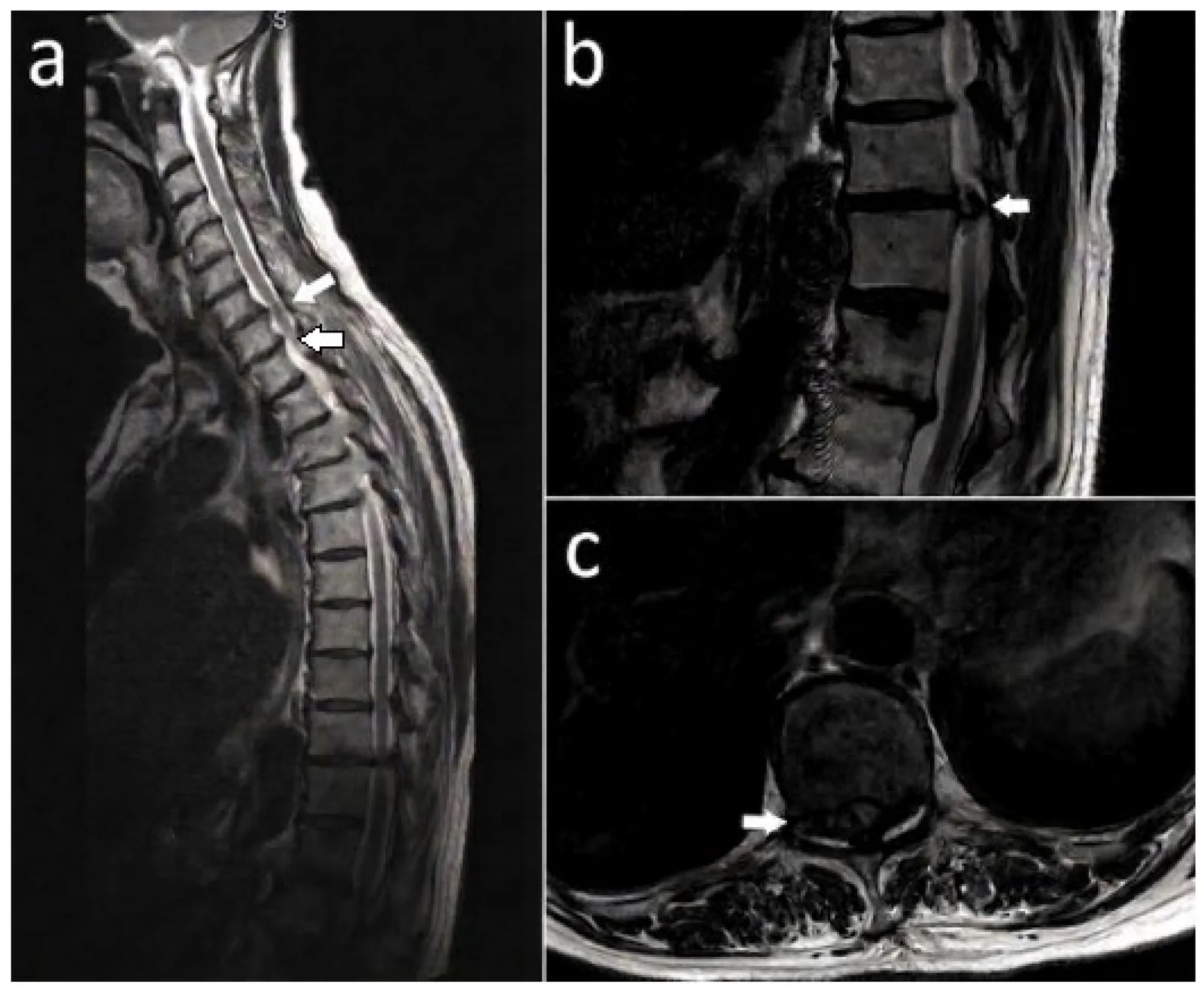
Given the discordance between the upper motor neuron signs and the absence of lumbar canal or cauda equina compression, and following CT characterization of the facet-associated lesion, dedicated cervical and thoracic MRI was performed. (**a**) Sagittal T2-weighted MRI of the cervical spine demonstrates multilevel spondylotic change but no significant central cervical canal stenosis or spinal cord compression at the levels indicated by the white arrows, making a cervical compressive cause of the neurological syndrome unlikely. (**b**) Sagittal T2-weighted MRI of the thoracic spine demonstrates mild-to-moderate canal narrowing at T9–T10 and severe stenosis at T10–T11, with marked deformation and compression of the thoracic spinal cord. The T10–T11 stenosis is multifactorial, resulting from a disc–osteophyte complex, facet-joint degeneration, ligamentum flavum thickening, and a 5-mm right posterolateral extradural cystic lesion abutting the facet/posterior elements (white arrow). (**c**) Axial T2-weighted MRI at T10–T11 confirms severe canal compromise and displacement of the thoracic spinal cord by the combined degenerative changes and right posterolateral extradural lesion (white arrow). In conjunction with the CT findings, the lesion was considered anatomically associated with the right T10–T11 facet/posterior-element region. Given its location, small cystic appearance, the absence of a definite expansile intraosseous lesion on CT, and the patient’s extensive degenerative facet disease, a degenerative facet or synovial cyst remained an important preoperative differential diagnosis. The lesion was considered a contributory component of the multifactorial T10–T11 stenosis rather than the sole cause of canal compromise. The severe cord compression provided a clear anatomical correlate for the patient’s progressive bilateral lower-limb weakness, sensory disturbance, gait deterioration, increased tone, and brisk reflexes. Following multidisciplinary spinal review, posterior T10–T11 decompression with excision of the cystic lesion was recommended because of the progressive neurological deterioration and severe spinal cord compression.

**Figure 4:**
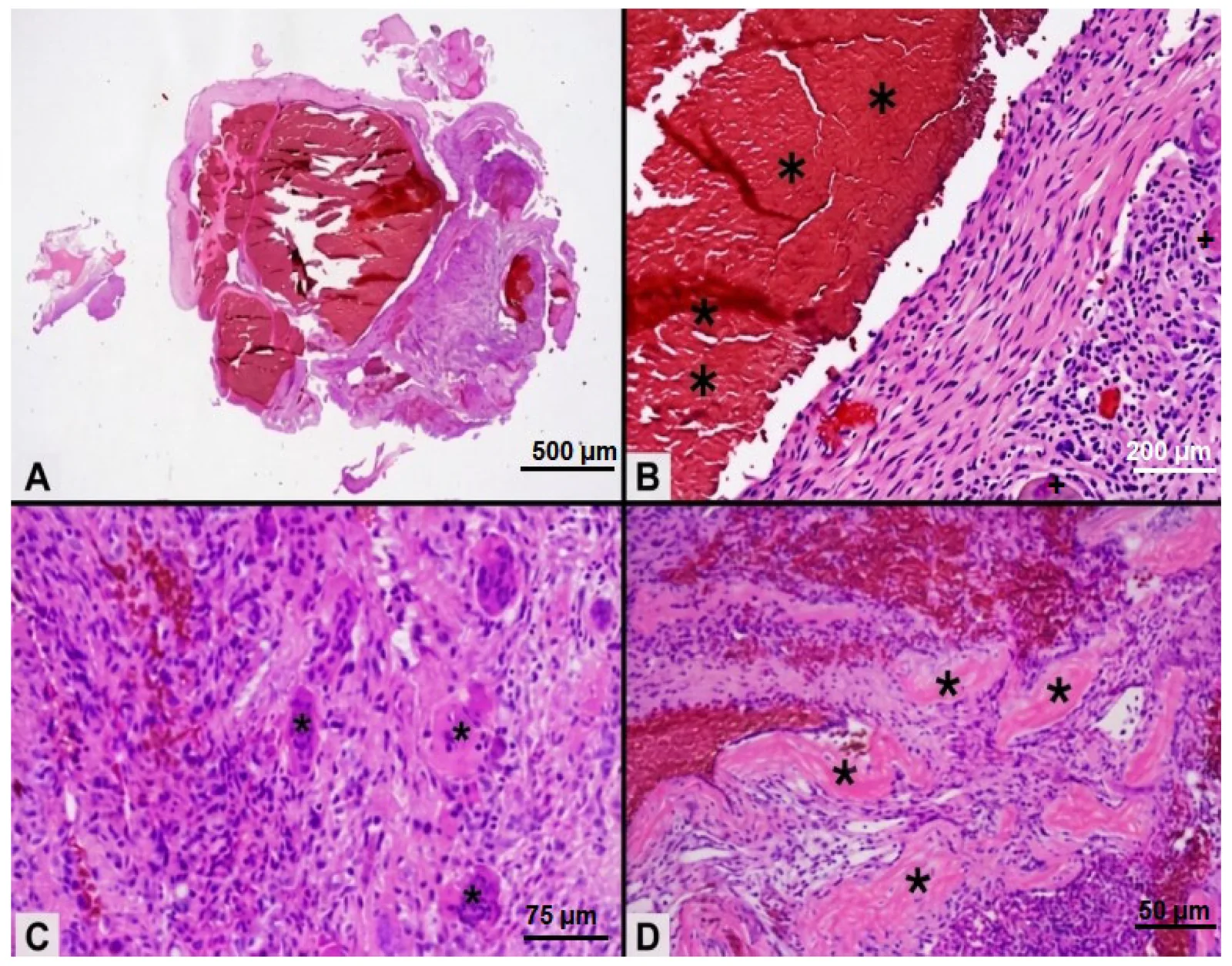
The patient underwent posterior T10–T11 decompression through a standard posterior midline approach, including laminectomy, excision of the ligamentum flavum, careful dissection, and complete removal (piecemeal) of the right posterolateral/facet-region extradural lesion. Adequate spinal cord decompression was confirmed intraoperatively, without dural tear, cerebrospinal fluid leakage, or neurological complication. The excised lesion was anatomically associated with the right T10–T11 facet-joint/posterior-element region. On imaging, the lesion appeared as a small right posterolateral extradural cystic lesion abutting the T10–T11 facet/posterior elements. Intraoperatively, a cystic lesion was identified in the corresponding region and was clearly distinct from the ligamentum flavum. The facet was preserved, and no facetectomy was performed. Although anatomically facet-associated, the precise tissue of origin (facet joint, adjacent bone, or periarticular soft tissue) could not be definitively established from the imaging and intraoperative findings. Postoperatively, spinal stability was assessed clinically and with dynamic radiographic assessment, with no evidence of pathological segmental instability at T10–T11. No additional stabilisation procedure was therefore required. The lesion was submitted for histopathological examination. All panels show haematoxylin and eosin (H&E)-stained sections. (**A**) Low-power image demonstrates the overall lesion architecture, comprising multiple irregular blood-filled cystic spaces separated by fibrovascular septa (×4). Physical scale bar: 500 μm. (**B**) Intermediate-power image shows fibrovascular septa containing an osteoclast-like multinucleated giant cell (black cross) and extensive haemorrhagic areas (black asterisks) (×10). Physical scale bar: 200 μm. (**C**) Higher-power image demonstrates osteoclast-like multinucleated giant cells (asterisks) within a fibrovascular septum (×20). Physical scale bar: 75 μm. (**D**) Higher-power image shows reactive bone formation within the fibrovascular stroma (asterisks) adjacent to the haemorrhagic cystic spaces (×10). Physical scale bar: 100 μm. The cystic spaces lacked a true endothelial lining. The combination of blood-filled spaces, giant-cell-containing fibrovascular septa, haemorrhage, and reactive bone formation, without cellular atypia or malignant features, was consistent with an aneurysmal bone cyst (ABC). ABC-like morphology may occur secondarily in other lesions, and primary ABCs commonly harbour USP6 rearrangements [4]. The differential diagnosis includes a degenerative synovial/facet cyst, giant cell tumor, osteoblastoma, telangiectatic osteosarcoma, metastatic disease, and other vascular or mesenchymal lesions [4,5,6,7,8]. In this patient, USP6 rearrangement testing was not performed, and neither imaging nor intraoperative findings demonstrated a definitive primary intraosseous origin. Accordingly, the lesion is best described as a facet-associated cystic lesion with ABC morphology rather than a confirmed primary osseous ABC. At approximately 2-month follow-up, the patient was independently ambulatory without a walking aid and reported improved walking distance, balance, and confidence. Lower-limb motor function had improved from approximately 3/5 preoperatively to approximately 4/5 throughout the L2–S1 distribution bilaterally, although residual lower-limb sensory impairment remained. Saddle sensation had returned to normal, bowel function was normal, and the patient reported no urinary dysfunction. Post-void residual volume had decreased from 203 mL preoperatively to 150 mL at follow-up. Lower-limb hyperreflexia and bilateral Hoffmann signs persisted despite thoracic decompression. Longer-term clinical and radiological surveillance was considered appropriate because lesions demonstrating ABC morphology may recur [5,6,7,8]. Several limitations should be acknowledged. The T10–T11 stenosis was multifactorial, precluding determination of the independent contribution of the cystic lesion to cord compression and the neurological presentation. The absence of USP6 testing and a definitively demonstrated intraosseous origin limits definitive classification of the lesion. Follow-up was limited to approximately 2 months, precluding assessment of long-term neurological recovery or recurrence. Finally, persistent lower-limb hyperreflexia and bilateral Hoffmann signs indicate that not all upper motor neuron findings can necessarily be attributed to the T10–T11 pathology. This case illustrates that thoracic cord compression may rarely present as a CES-like syndrome [3]. When lumbar MRI does not explain the clinical findings and upper motor neuron signs are present, cervical and thoracic imaging should be considered. Likewise, a facet-region cystic lesion with atypical clinical, imaging, operative, or histopathological features should not automatically be assumed to be degenerative.

## Data Availability

No new datasets were generated or analyzed during the preparation of this manuscript. All relevant clinical, radiological, operative, and histopathological information supporting the findings of this report is contained within the manuscript. Additional patient-level data are not publicly available because of privacy and ethical considerations.

## Abbreviations

ABC—Aneurysmal bone cyst. CES—Cauda equina syndrome. H&E—Haematoxylin and eosin. MRC—Medical Research Council. MRI—Magnetic resonance imaging.
